# Enhanced Natural Strength: Lamiaceae Essential Oils and Nanotechnology in In Vitro and In Vivo Medical Research

**DOI:** 10.3390/ijms242015279

**Published:** 2023-10-17

**Authors:** Tomasz Kowalczyk, Anna Merecz-Sadowska, Mansour Ghorbanpour, Janusz Szemraj, Janusz Piekarski, Michal Bijak, Tomasz Śliwiński, Radosław Zajdel, Przemysław Sitarek

**Affiliations:** 1 Department of Molecular Biotechnology and Genetics, Faculty of Biology and Environmental Protection, University of Lodz, Banacha 12/16, 90-237 Lodz, Poland; tomasz.kowalczyk@biol.uni.lodz.pl; 2 Department of Economic and Medical Informatics, University of Lodz, 90-214 Lodz, Poland; anna.merecz-sadowska@uni.lodz.pl (A.M.-S.); radoslaw.zajdel@uni.lodz.pl (R.Z.); 3 Department of Medicinal Plants, Faculty of Agriculture and Natural Resources, Arak University, Arak 38156-8-8349, Iran; m-ghorbanpour@araku.ac.ir; 4 Department of Medical Biochemistry, Medical University of Lodz, 92-215 Lodz, Poland; janusz.szemraj@umed.lodz.pl (J.S.); tomasz.sliwinski@biol.uni.lodz.pl (T.Ś.); 5 Department of Surgical Oncology, Medical University in Lodz, 93-513 Lodz, Poland; janusz.piekarski@umed.lodz.pl; 6 Biohazard Prevention Centre, Faculty of Biology and Environmental Protection, University of Lodz, Pomorska 141/143, 90-236 Lodz, Poland; michal.bijak@biol.uni.lodz.pl; 7 Department of Medical Biology, Medical University of Lodz, Muszyńskiego 1, 90-151 Lodz, Poland

**Keywords:** antioxidant effect, anti-inflammatory effect, wound healing, anti-aging, aromatic plants, Lamiaceae essential oils, nanoparticles, patents

## Abstract

The Lamiaceae is one of the most important families in the production of essential oils known to have a wide spectrum of biological activity. Recent research has highlighted the dermatological capabilities of various Lamiaceae essential oils, which appear to offer potential in free radical scavenging and anti-inflammatory activity. Some have also been extensively studied for their tissue remodeling and wound-healing, anti-aging, anti-melanogenic, and anti-cancer properties. Certain Lamiaceae essential oils are promising as novel therapeutic alternatives for skin disorders. This potential has seen substantial efforts dedicated to the development of modern formulations based on nanotechnology, enabling the topical application of various Lamiaceae essential oils. This review provides a comprehensive summary of the utilization of various essential oils from the Lamiaceae family over the past decade. It offers an overview of the current state of knowledge concerning the use of these oils as antioxidants, anti-inflammatory agents, wound-healers, anti-aging agents, anti-melanogenic agents, and anticancer agents, both alone and in combination with nanoparticles. Additionally, the review explores their potential applicability in patents regarding skin diseases.

## 1. Introduction

**Figure 1:**
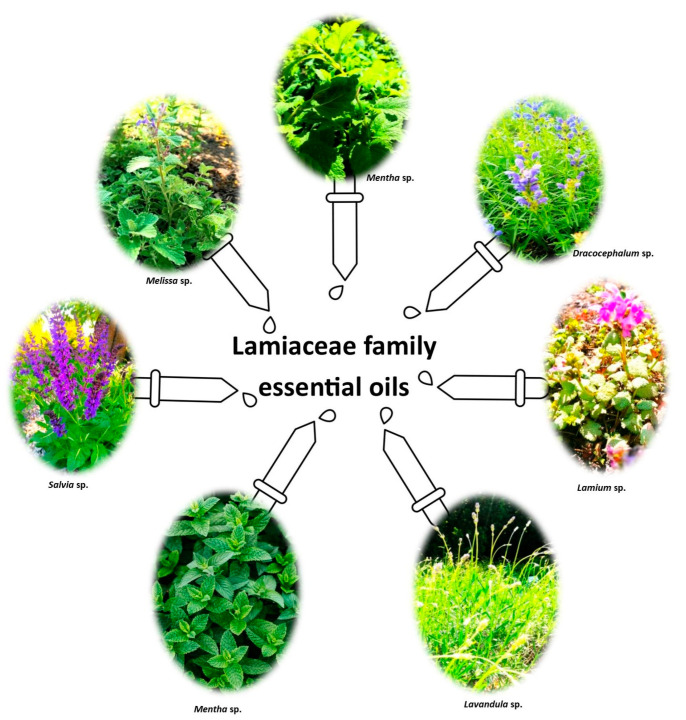
General overview of the Lamiaceae family.

The Lamiaceae is one of the largest flowering plant families in the world with more than 7000 species and about 250 genera. The family members are cosmopolitan, being found in the Americas and Mediterranean regions, but also in southern Africa, Australia and Asia, and comprise herbs, herbaceous plants, shrubs, and tree species [1,2,3]. The family itself is commonly known as the mint family, or as chun xing ke, irumba-hare, irumbahe, or lumbase nilcols. Due to their aromatic properties, the Lamiaceae have great economic importance in, inter alia, cosmetics, foods and modern medicine [4,5], particularly the genera *Mentha*, *Ocimum*, *Salvia*, *Clerodendrum*, and *Plectranthus* [1,6,7]. Example genera from the Lamiaceae family are shown in Figure 1.

The aromatic plants in this family are also rich in essential oils (EOs), i.e., complex mixtures of low molecular weight (usually less than 500 daltons) compounds [8,9]. EOs consist of various organic compounds [6,10,11] whose functional groups possess certain properties. The most common EOs are derived from terpenoids and phenylterpenoids, of which the most common are monoterpenes [6,12]. They are synthesized by all tissues and stored in secretory cells, epidermal cells, or glandular hairs [6,8,13]. They can be extracted from any plant organ, such as buds, stems, twigs, leaves, roots, wood, bark, flowers, fruits, and seeds. Their chemical composition depends not only on the species of plant or plant tissue from which they are extracted, but also on the climate, soil composition, vegetative cycle, or age, and even the harvest time. A key role is also played by the extraction method, which can have a significant influence on the quality of EOs [14,15,16]. Among other things, EOs protect plants against fungi and bacteria, defend against insects and herbivorous animals, and attract pollinators to facilitate seed and pollen dispersal [11,17,18]. EOs also have important antioxidant, anti-inflammatory, antibacterial, antifungal, and antiviral properties [19,20,21,22,23].

Skin diseases can be associated with oxidative stress and inflammation. The normal functions of proteins, lipids, and DNA are destabilized by oxidative stress sustained by free radicals. This can also affect a number of signaling pathways associated with inflammation, thereby affecting cell and tissue homeostasis [24,25]. In addition, inflammation is characterized by the production of pro-inflammatory cytokines and chemokines, leading to pain, redness, and swelling of the affected tissue. These have been found to be relieved by EOs, which has been attributed to the presence of antioxidant and anti-inflammatory compounds, such as terpenes, and, especially, monoterpenes [5,6,26].

This review presents findings on the biological activity of EOs from the Lamiaceae family from the last 10 years in the context of various skin lesions; it discusses the antioxidant, anti-inflammatory, wound-healing, anti-aging, anti-melanogenic, and anti-cancer properties of the EOs, as well as the mechanisms of action of their active ingredients. It also discusses new methods of nanoparticle delivery into cells, and reviews patent applications as an example of their industrial use in cosmetology.

## 2. Sources and Search Criteria

The studies were identified through searches in the electronic databases Web of Science, Scopus, PubMed, and Google Scholar. The following keywords and phrases were used: “aroma plants from Lamiaceae”, “antioxidant activity of essential oils”, “anti-inflammatory effect of essential oils”, “wound healing activity of essential oils”, “anti-aging effect of essential oils”, “anti-melanogenic effect of essential oils”, “anti-cancer activity of essential oils”, “cytotoxic effect of essential oils”, “skin diseases and essential oils”, “in vitro activity of essential oils”, and “nanoparticles with essential oils”. In total, 210 relevant articles were obtained after extraction and analysis by combining the above keywords/phrases and inclusion criteria. However, after selection, their number decreased.

To be included, articles had to encompass the characteristics of Lamiaceae essential oils with a primary focus on the following properties: (a) their antioxidant potential, (b) anti-inflammatory qualities, (c) wound-healing properties, (d) anti-aging attributes, (e) anti-melanogenic effects, (f) anticancer capabilities, (g) their integration with nanoparticles, and (h) patent-related documentation.

## 3. Antioxidant Potential of Essential Oils from Plants Belonging to the Lamiaceae Family

Free radicals are molecules characterized by an unpaired electron in their valence shell or outer orbit, which makes them highly reactive. Reactive oxygen species (ROS) and reactive nitrogen species (RNS), such as the hydroxyl radical, superoxide anion radical, singlet oxygen, hydrogen peroxide, nitric oxide, and peroxynitrite, are harmful to the skin. An imbalance between the production of free radicals and the ability of the body to neutralize them is defined as oxidative stress. The skin, a barrier between the body and the external environment, is subjected to oxidative stress caused by exogenous agents, such as UV radiation or atmospheric pollutants. In addition, some free radicals are also produced as oxidative metabolism byproducts in the mitochondria. The impact of reactive species on the skin can be significant, leading to various skin problems [24,27].

Free radicals can cause damage to cellular components, such as collagen and elastin, which are key dermal structural proteins. Collagen can only be cleaved by metalloproteinase 1 (MMP1) and then completely degraded by MMPs 2, 3 and 9. ROS can impact on MMP-1 activity indirectly through the modulation of signaling networks that contribute to its expression, including mitogen-activated protein kinase (MAPK) signaling [28]. Oxidative stress also directly impacts collagen, liberating small peptides containing 4-hydroxyproline by superoxide anion protein fragmentation. In the presence of oxygen, hydroxyl radicals release small peptides comprising proline or 4-hydroxyproline residues; however, in the absence of oxygen, the radicals trigger their polymerization and new dityrosine or disulfate cross-link formation [29]. Moreover, tropoelastin is sensitive to ROS, indicating that ROS may induce elastotic changes or deposition of fragmented elastic fibers [30]. In addition, it has been shown that direct oxidation of elastin in the presence of hydrogen peroxide results in transformation of the cross-links of desmosine and isodesmosine to oxodesmosine and isooxodesmosine [31], resulting in changes in protein conformation and unfolding, leading to aging and changes in skin mechanical properties.

Free radicals can cause damage to melanocytes, i.e., the cells responsible for producing the pigment melanin. This damage can lead to a reduction in melanin production, potentially causing uneven skin pigmentation. There is strong evidence for the role of oxidative stress as an important factor in the onset and progression of vitiligo, a depigmentary disease characterized by the loss of melanocytes in the epidermis, with a global prevalence of approximately 0.5% [32,33]. Patients have been found to demonstrate elevated levels of malondialdehyde, a marker of oxidative damage, and decreased levels of the antioxidants superoxide dismutase and reduced glutathione [34]. Other data indicate high superoxide dismutase activity in the serum of vitiligo patients [35], as well as very high levels of hydrogen peroxide in the epidermis and reduced catalase levels and activity [36]. Elevated levels of hydrogen peroxide inactivate methionine sulfoxide reductase A and B, and thioredoxin/thioredoxin reductase, thus inducing oxidative stress and melanocyte death, resulting in vitiligo [37,38]. It may also oxidize proopiomelanocortin-derived bioactive peptides, like adrenocorticotropic hormone (ACTH) and α-melanocortin (α-MSH), both of which have antioxidant effects on human pigment cells and promote their survival. These modifications can lead to skin discoloration [39,40,41]. 

Free radicals can cause DNA damage and modulate signaling pathways, thus increasing the risk of skin cancer. The most important skin tumors are epithelial tumors, basal cell carcinoma, and squamous cell carcinoma [42]. The highly reactive hydroxyl radical reacts with DNA by binding to the double bonds of DNA bases. Addition to the C5-C6 double bond of pyrimidines leads to the formation of C5-OH and C6-OH adduct radicals. Pyrimidine radicals yield various products by different mechanisms, including cytosine or thymine glycol. Hydroxyl radicals add to the C4, C5, and C8 positions of purines, generating OH adduct radicals and resulting in the formation of C4-OH, C5-OH, and C8-OH adduct radicals. C4-OH and C5-OH adduct radicals may reconstitute the purine, whereas C8-OH adduct radicals lead to 8-hydroxyguanine formation. 8-hydroxyguanine may cause DNA strand breaks by reacting with 2′-deoxyribose present in DNA and H abstraction. When reacting with DNA, the hydroxyl radicals can also abstract the H atom from the methyl group of thymine, resulting in the formation of an allyl radical, yielding 5-hydroxymethyluracil and 5-formyluracil for each of the C-H bonds of 2-deoxyribose [43].

ROS also modulate signaling pathways. For example, ROS may favor the development of skin tumors by regulating the activation of NF-κΒ. On the one hand, ROS activate NF-κΒ via oxidation of LC8, a component of the cytoplasmic dynein complex. Briefly, oxidized LC8 dissociates from I-κBα, the major NF-κB inhibitor protein, resulting in NF-κB activation; this has anti-apoptotic effects on melanoma cells and encourages tumor growth. However, ROS also inhibit NF-κΒ via the oxidation of thioredoxin, a regulatory protein. In its reduced state in the nucleus, thioredoxin elevates NF-κB activity by promoting its binding to DNA. Moreover, chronic oxidative stress may limit DNA binding via direct oxidation of NF-κB heterodimers [44]. Other transcription factors, including hypoxia-inducible factor-1 (HIF-1) and p53, also undergo oxidative modification, which affects their DNA binding properties and, thus, gene transcription [45].

The harmful effects of free radicals are countered by antioxidants, which neutralize free radicals by donating an electron. Each antioxidant has its own chemical structure and unique properties. High levels of antioxidants are found in plants. Their antioxidant effect has been widely studied and documented as having numerous benefits for the skin. Plant-derived antioxidants help to protect the skin from skin aging, uneven skin pigmentation, and other skin conditions, including cancer [46,47,48]. The antioxidant properties of EOs from the Lamiaceae have been examined in previous studies [6,49].

## 4. Anti-Inflammatory Potential of Essential Oils from Plants Belonging to the Lamiaceae Family

**Figure 2:**
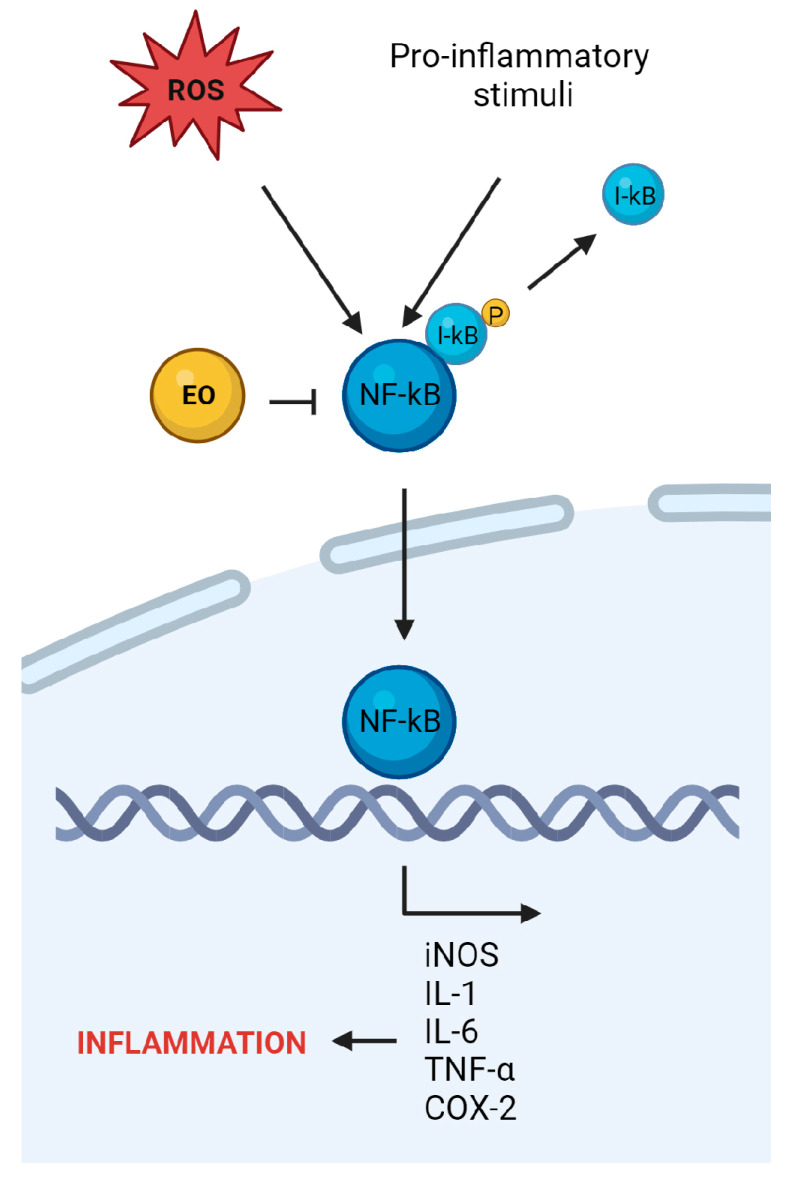
Schematic presentation of anti-inflammatory properties of EOs derived from Lamiaceae family. Pro-inflammatory stimuli and ROS may increase the inflammatory response within the cells, leading to increased expression of NF-κβ. EOs may counteract inflammation and consequently decrease damage to the cells (created by BioRender.com, https://www.biorender.com/ accessed on 26 July 2023); iNOS—inducible nitric oxide synthase, IL—interleukin, TNF-α—tumor necrosis factor α, COX-2—cyclo-oxygenase 2. Sharp arrows (→) indicate stimulation while blunt arrows (┴) indicate inhibition.

The immune reaction is crucial for defense against pathogens. However, prolonged inflammation may trigger downstream signaling cascades, leading to immune-related proteins release that intensifies tissue damage (Figure 2). 

A dysregulated immune response may result in chronic inflammatory skin diseases. These range from mild ailments, like acute rashes with itching and redness, to chronic conditions, like psoriasis or atopic dermatitis (AD) [50,51]. Psoriasis is manifested by red, thickened skin, and skin scales due to keratinocyte hyperproliferation. It affects 2 to 3% of the population. Characteristic features of psoriasis are infiltration of cytotoxic CD8+ lymphocytes into the epidermis, infiltration of the dermis and subcutaneous tissue by CD4+ T cells, macrophages and neutrophils, activation of mast cells, and abnormal development of blood vessels. Psoriatic lesions display enhanced production of pro-inflammatory factors, including TNF-α, interferon-gamma (IFN-γ), IL-6, IL-8, IL-12, IL-17, IL-18, and reduced production of anti-inflammatory factors, including IL-4 and IL-10. AD is characterized by an age-dependent distribution of cutaneous lesions with eczema-like morphology. It affects 10 to 20% of children and 3% of adults. AD is characterized by, inter alia, infiltration of CD4+ lymphocytes, mast cells, eosinophils, histiocytes, and Langerhans cells. AD has an acute phase characterized by the enhanced production of TNF-α, IL-4, IL-5, IL-13, and immunoglobulin E (IgE), and a chronic phase with enhanced production of TNF-α, IFN-γ, IL-8, and IL-12 [52,53,54].

The anti-inflammatory effects of the Lamiaceae EOs in cell and animal models are shown in Table 1 and Table 2.

## 5. Wound-Healing Potential of Essential Oils from Plants Belonging to the Lamiaceae Family

The human body undergoes a natural process of wound healing, which involves four distinct and highly coordinated phases: hemostasis, inflammation, new tissue formation, and tissue remodeling. It is crucial that these phases occur in the correct order, at specific times, and with optimal intensity. In hemostasis, the first stage, the wound is closed by clotting. Collagen is exposed during the formation of the wound, which triggers the clotting cascade and initiates the inflammatory phase. This leads to the release of a cellular distress signal, and the first responders to the area are neutrophils. Monocytes are also drawn to the area and transform into macrophages. Activation of these inflammatory cells is critical, particularly for the macrophage, which is necessary for the transition into the proliferative phase. The proliferative phase involves key steps, such as epithelialization, angiogenesis, granulation tissue formation, and collagen deposition. Finally, the remodeling phase is focused on the deposition of collagen in an organized and orderly network [89,90].

It was found that 8.2 million individuals reported acute and chronic wounds with or without infections. It was estimated that the cost of treating acute and chronic wounds, including infection management, was between USD 28.1 billion and USD 96.8 billion. Plant-derived molecules provide an alternative method of promoting wound healing. The literature reports strong evidence of the anti-inflammatory, antioxidant, and antimicrobial effects of EOs, which are crucial in treating chronic wounds [91,92].

Avola et al. conducted a study to investigate the wound-healing potential of *Origanum vulgare* EO on human keratinocytes using a scratch test. Carvacrol was found to be the major component of the EO, followed by thymol, p-cymene, and linalool, while other compounds were present in trace amounts. Treatment with 25 µg/mL concentration of the EO showed a significant improvement in cell motility and wound healing 72 h after injury, compared to the untreated control. The study suggests that *O. vulgare* EO has the potential to support and enhance the wound-healing process.

A study carried out by Farahpour et al. revealed that *Salvia officinalis* EO contains cis-thujone, camphor, trans-thujone, and 1,8-cineole as its main compounds. Topical application of the 2% and 4% (*w*/*w*) EO on mice with surgically created wounds with 5 mm diameter on the dorsal surfaces accelerated the wound-healing process by shortening the inflammatory phase, promoting cellular proliferation, re-vascularization, collagen deposition, and re-epithelialization compared to the control group. The study also found increased mRNA levels of fibroblast growth factor 2 (FGF-2) and vascular endothelial growth factor (VEGF), upregulation of cyclin-D1 and Bcl-2, and reduced expression levels of IL-6, IL-1β, and TNF-α in animals treated with *S. officinalis* essential oil on days 3, 7, and 14. Therefore, the study concluded that the EO of *S. officinalis* has antioxidant, anti-inflammatory, and growth-promoting properties, making it effective in accelerating wound healing [93].

Chabane et al. conducted a study that identified the primary constituents of *Teucrium polium* EO in the following order: β-pinene, germacrene, α-pinene, myrcene, limonene, bicyclogermacrene, trans-β-guaiene, spathulenol, and β-bourbonene. The EO was incorporated into petroleum jelly to create a 10% concentration ointment, which was then topically applied once daily for 16 days to surgically created excisional 2.5 diameter wounds on the skin of the lumbar region of rabbits. In vivo experiments demonstrated that 10% *T. polium* EO ointment accelerated the wound-healing process compared to the control group. These findings support the use of *T. polium* EO as a potential treatment for wounds [94].

Napoli et al. report that the EO of *Pistacia vera* consist of a complex mixture of phytochemicals, including numerous volatile compounds, as well as several non-volatile constituents, primarily tri- and tetraterpenoids. The potential of *P. vera* EO in wound healing was examined using a rabbit excision wound model. A 2.5 cm diameter circle was excised from the lumbar region to create an excisional wound, which was treated immediately. The EO was combined with petroleum jelly to produce a topical ointment at a final concentration of 5%, and 0.5 g of the ointment was applied once daily per rabbit for 16 days. The EO demonstrated capabilities in promoting wound healing, indicating their potential use in modern therapy [95].

The effects of an ointment prepared from *Zataria multiflora* EO on infected wounds were evaluated in a study by Farahpour. The EO was analyzed and found to contain 29 compounds, which accounted for 99.6% of its total composition. In the study, a full-thickness excisional skin wound 7 mm in size was surgically created in each mouse and inoculated with a suspension containing *Pseudomonas aeruginosa* and *Staphylococcus aureus*. The mice were treated with an ointment containing 4% *Z. multiflora* essential oil for 21 days. The results showed that the topical application of *Z. multiflora* EO significantly reduced the total bacterial count and wound area, as well as the expression of IL-1β and TNF-a, compared to the control groups on all days. In addition, it increased the expression of TGF-β, IL-10, insulin-like growth factor-1 (IGF-1), FGF-2, and VEGF, as well as angiogenesis, fibroblasts, fibrocytes, epithelialization ratio, and collagen deposition, and improved the antioxidant status compared to the control group. These findings suggest that *Z. multiflora* EO can accelerate the healing process of infected wounds by shortening the inflammatory phase and increasing the proliferative phase, and could be a potential treatment for wound healing [88].

A study was conducted by Modaressi et al. to assess the wound-healing effects of ointments containing *Mentha piperita* EO in infected mouse models. A circular wound with a diameter of 7 mm was created on the dorsal surfaces of the mice, and each wound was inoculated with strains of *Staphylococcus aureus* and *Pseudomonas aeruginosa*. Topical administration of 0.5 g of each ointment containing 2%, 4%, and 8% *M. piperita* (*w*/*w*) was performed once per day, 24 h after bacterial colonization, for 16 days. The results showed an increase in fibroblast migration, collagen synthesis, and re-epithelization in treated animals with EO. These findings suggest that *M. piperita* EO could be used as a potential treatment for infected wounds [96].

**Figure 3:**
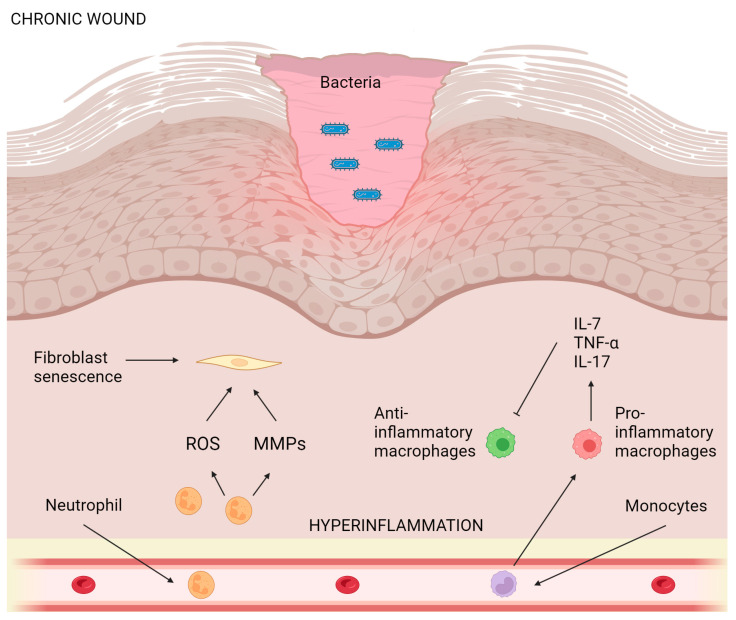
Schematic representation of wound healing after applying essential oils from the Lamiaceae family. Chronic wounds often display persistent inflammation, biofilm-associated infections, hyperproliferative epidermis, fibroblast senescence, increased levels of MMPs, and impaired cellular migration. (Created by BioRender.com https://www.biorender.com/ accessed on 26 July 2023). Sharp arrows (→) indicate stimulation while blunt arrows (┴) indicate inhibition.

Some common bacteria that can affect the skin include *Staphylococcus aureus*, *Streptococcus pyogene*, and *Pseudomonas aeruginosa* [97,98]. Skin and soft tissue infections caused by these microorganisms are a significant health problem (Figure 3). Moreover, cutaneous mycoses of the skin are often subject to dermatological disorders. Fungal skin infections may be classified as dermatophytoses or dermatomycoses. The former is caused by agents belonging to the genera *Epidermophyton*, *Microsporum*, and *Trichophyton*. The latter refers to skin infections caused by other types of fungi, with *Candida* spp. being among the most commonly encountered. Lamiaceae EOs have been found to have antimicrobial effects against *Staphylococcus aureus*, *Streptococcus pyogenes*, and *Pseudomonas aeruginosa*, as well as *Epidermophyton*, *Microsporum*, *Trichophyton*, and *Candida* [6,23].

## 6. Anti-Aging Potential of Essential Oils from Plants Belonging to the Lamiaceae Family

Aging is a natural process that affects all layers of the skin, caused by the breakdown of its components. ROS and degradative enzymes play a major role in the aging process. Skin damage leading to aging is typically slowed or restored using anti-aging products. Some plants have been found to protect the skin matrix by inhibiting enzymatic degradation, promoting collagen synthesis, and scavenging free radicals. Certain plants can also enhance skin elasticity and tightness. However, further research is needed to determine the anti-aging potential of EOS, including their ingredient concentrations, formulation, safety, and durations of effect. Various members of the Lamiaceae have been reported to have anti-aging effects [99,100].

Laothaweerungsawat and colleagues conducted a study on the potential of *Origanum vulgare* EO as a skin-ageing retardant. The major component of the EO was found to be carvacrol. The study evaluated its anti-skin-ageing properties by measuring its ability to inhibit collagenase, elastase, and hyaluronidase. The EO was found to demonstrate significantly better anti-skin-ageing activity compared to ascorbic acid, with inhibitory concentrations of 67, 25, and 4 µg/mL against collagenase, elastase, and hyaluronidase, respectively. Based on these findings, the EO was suggested to be a promising natural ingredient for use in the cosmetic industry to prevent skin ageing [101]. Similarly, *Mentha viridis* EO demonstrated anti-elastase potential; the oil was found to contain 28 compounds, including carvone, 1,8-cineole, and terpinen-4-ol [102].

Lin et al. examined the protective effects of *Pogostemon cablin* EO against UV-induced skin photoaging in mice. The major component of the EO was patchouli alcohol, followed by β-gurjunene and β-guaiene, and other minor components. The oil was primarily composed of terpenoids, with monoterpenes and sesquiterpenes comprising over 50%. During the experiment, the dorsal skin of the mice was treated with EO for two hours prior to UV exposure. The protective effects of the oil were evaluated using macroscopic and histological assessments, skin elastic tests, collagen content measurements, and assays of biochemical indicators, such as malondiaidehyde (MDA) content, superoxide dismutase (SOD), glutathione peroxidase (GSH-Px), and catalase (CAT). The results showed that, compared to the UV-exposed groups, the application of EO, particularly at doses of 6 mg/mouse and 9 mg/mouse, significantly inhibited the formation of skin wrinkles, increased skin elasticity, and raised collagen. Furthermore, the application of 6–9 mg/mouse EO also reduced epidermal thickness and prevented the disruption of collagen and elastic fibers induced by UV exposure. The application of EO also decreased the content of MDA and significantly upregulated the activities of SOD, GSH-Px, and CAT. The results indicate that EO has the potential to prevent photoaging by maintaining the structural integrity of the skin and exerting various anti-oxidative properties. Therefore, the authors suggest that EO should be considered as a potential therapeutic agent for preventing photoaging [103].

## 7. Anti-Melanogenic Potential of Essential Oils from Plants Belonging to the Lamiaceae Family

Melanin biosynthesis takes place in melanocytes, dendrite-shaped cells in the epidermis that distribute melanin pigments to neighboring keratinocytes. Melanocytes are essentially associated with keratinocytes to form the epidermal melanin unit (EMU) with a ratio of one melanocyte to ~40 keratinocytes. This cross-link between cells is fundamental for the transfer of melanin pigments to keratinocytes, conferring skin color and protecting against UV radiation. However, many factors, such as intracellular pH, prolonged UV exposure, and aging, lead to abnormal melanin production and its accumulation in the skin. For example, in skin, UV exposure increases the expression of MSH (melanocyte stimulating hormone) as well as the number of melanocytes, leading to irregular melanin distribution. In addition, it causes deregulation of some pro-melanogenic factors and their receptors and results in dysfunction of some proteins and enzymes in the skin. This leads to an over-production of melanin, which results in hyperpigmentation. 

Skin hyperpigmentation can be controlled by various methods ranging from prevention to correction. The first preventative approach to treat hyperpigmentation is to inhibit the catalysis of tyrosinase—the first enzyme involved in melanogenesis—through the use of UV filters that form a physical barrier to UV radiation. A second approach is to control the over-production of melanin by inhibiting melanogenesis within melanocytes, and subsequently to inhibit the transfer of melanin pigments from melanocytes to keratinocytes. A final approach is to correct the color and remove excessive melanin in the epidermis by desquamation and epidermal renewal. Of all these approaches, tyrosinase inhibition is the most targeted method for the treatment of hyperpigmentation. Tyrosinase is a copper-containing enzyme that catalyzes the hydroxylation of L-tyrosine into dihydroxy-phenylalanine (L-DOPA) and the oxidation of L-DOPA into DOPAquinone. This reaction represents the first step of melanogenesis.

The anti-melanogenic properties of EOs from the Lamiaceae, which were evaluated based on cellular models, are presented below [104,105,106,107].

El Khoury examined the effects of two *Origanum* essential oils on the melanogenic activity of B16-F1 murine melanocytes. The main component of these oils, carvacrol, was also investigated. The essential oils of *Origanum syriacum* and *Origanum ehrenbergii* were found to significantly reduce melanin levels at 40 g mL^−1^. Similarly, carvacrol reduced melanin levels at 45 g mL^−1^. These results suggest that the oils and carvacrol have anti-melanogenic properties. The authors suggest that carvacrol may function as a competitive inhibitor of tyrosinase, thereby inhibiting the oxidation of tyrosine and causing disruption of melanogenesis [108].

Chou et al. examined the potential anti-melanogenic effects of *Glechoma hederacea* EO. The main components of the oil were trans-3-pinanone, 4,5,6,7-tetrahydro-5-isopropenyl-3,6-beta-dimethyl-6-alpha-vinylbenzofuran, β-caryophyllene, and spathulenol. It was found that the EO significantly reduced melanin production and tyrosinase activity in B16 cells stimulated with α-MSH. The cells were treated with various concentrations of the EO (2.5, 5, 10, and 20 μg/mL) for 72 h. The study also suggested that the anti-melanogenic effects of the EO might be due to its strong antioxidant properties, as evidenced by reduced levels of cellular oxidants and MDA, as well as improved activities of glutathione peroxidase (GPx) and SOD in α-MSH-stimulated B16 cells [55].

## 8. Anti-Cancer Potential of Essential Oils from Plants Belonging to the Lamiaceae Family

Research has demonstrated that EOs exhibit anticancer properties via diverse mechanisms, such as cancer preventative mechanisms, direct effects on established tumor cells, and interaction with the microenvironment. The essential oils exhibit antimutagenic, antiproliferative, antioxidant, and detoxifying capabilities.

One mechanism involves the direct blockage of the mutagen from entering the cell. Additionally, EOs have been shown to reduce the expression of phase I enzymes, such as cytochrome C, thereby preventing mutagen formation, while enhancing the activity of phase II enzymes, such as glutathione S-transferase, uridine 5′-diphospho-glucuronosyltransferase, quinone reductase, and epoxide hydrolase, for increased detoxification. By binding ROS and forming reactive phenoxy radicals, EOs can prevent oxidative damage and prevent cancer, while simultaneously increasing the activity of antioxidant enzymes, including CAT, SOD, GPx, and glutathione (GSH). EOs induce apoptosis by disrupting mitochondrial membrane potential, resulting in an increase in ROS and a decrease in GSH, the release of cytochrome C, and subsequent perturbation of the Bcl/Bax ratio. This triggers the activation of caspase 3 and caspase 9, and the cleavage of poly (ADP-ribose) polymerase (PARP). Additionally, essential oils inhibit the mechanistic target of rapamycin (mTOR) and protein pyruvate dehydrogenase kinase 1 (pPDK1), resulting in the dephosphorylation of protein kinase B (PKB) and activation of caspase activity, while deactivating murine double minute 2 (mdm2). This leads to an increase in p21, which further initiates caspase activity and induces G1/S phase cell cycle arrest. Moreover, EOs cause a decrease in cyclin-dependent kinase 7 (CDK7), blocking the CDK1/cyclin complex, and leading to G2/M phase cell cycle arrest [109,110].

**Figure 4:**
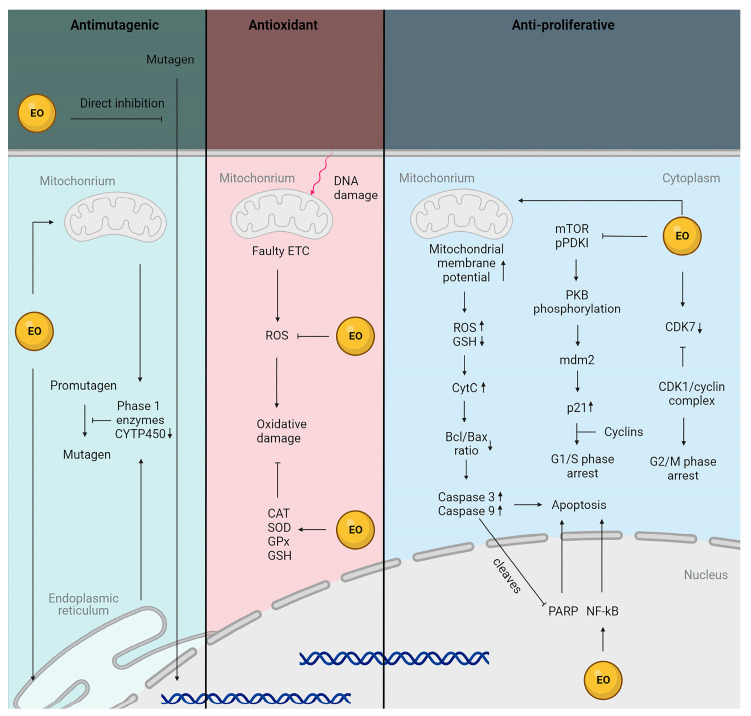
Schematic presentation of anticancer properties of essential oils derived from Lamiaceae family. EOs demonstrate antimutagenic, antioxidant, and antiproliferative properties by influencing multiple pathways within cancer cells. (created by BioRender.com https://www.biorender.com/ accessed on 26 July 2023). Sharp arrows (→) indicate stimulation while blunt arrows (┴) indicate inhibition.

The inhibitory effects of essential oils derived from the Lamiaceae family on melanoma cells are presented in Figure 4 and Table 3.

Govindaraju and Arulselvi investigated the potential antiproliferative activity and cytotoxicity of *Coleus aromaticus* leaf EO and its purified constituent, carvacrol, on human melanoma A375 cells. The effect of carvacrol on cell cycle arrest, DNA fragmentation, and apoptosis was also evaluated by examining the cleavage of poly (ADP-ribose) polymerase (PARP) and Bcl-2 gene expression. A375 cells were treated with carvacrol for 24 h, which resulted in the inhibition of cell growth. Carvacrol reduced the viability of A375 cells in a concentration-dependent manner, with an IC_50_ value of 40.41 μg/mL. Additionally, carvacrol was found to inhibit the growth of A375 cells by inducing apoptosis, as demonstrated by acridine orange/ethidium bromide staining and flow cytometry analyses. The treatment of A375 cells with carvacrol also led to the cleavage of PARP and a decrease in Bcl-2 gene expression, which further supported the induction of apoptosis. The findings suggest that carvacrol may induce apoptosis by directly activating the mitochondrial pathway, which could be a crucial mechanism underlying its anticancer effect [124].

## 9. Nanotechnology as a Strategy for Precise Delivery of Lamiaceae Essential Oils in Skin Diseases

**Figure 5:**
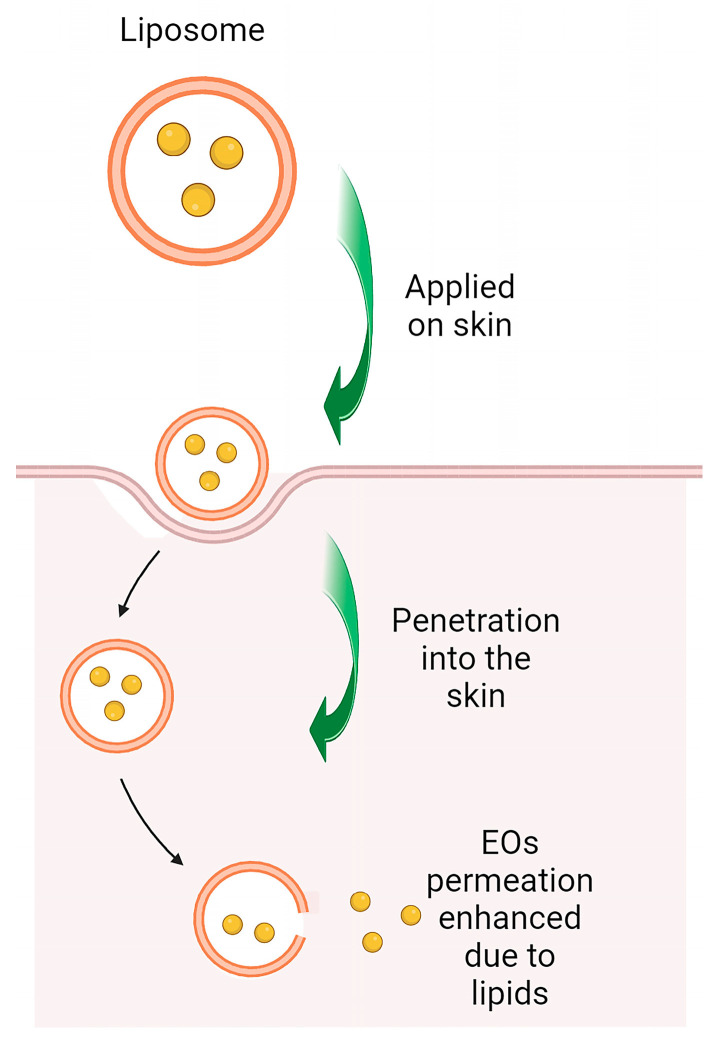
Schematic presentation of essential oils derived from Lamiaceae family permeation enhanced due to lipid nanoparticles. Liposomes act as an intracellular delivery system, facilitating molecules deposition into the stratum corneum. (Created by BioRender.com https://www.biorender.com/ accessed on 26 July 2023). Sharp arrows (→) indicate stimulation.

Percutaneous penetration of active molecules is currently a major area of research. Although such effective methods would increase accessibility and enhance therapeutic efficacy, molecule delivery through the skin is challenging due to its low permeability [125,126]. Despite the development of various methods that improve skin penetration, penetration of therapeutic agents is often aggressive and can lead to irreversible damage [126,127]. The use of nanoparticles as carrier systems represent an alternative approach to currently classical technologies, with minimal damage to the skin’s natural barrier function and delivery of compounds in a precise manner [128,129]. They have many advantages as topical drug delivery systems, such as greater molecule deposition in the target region, providing increased physicochemical stability for the molecules loaded in the nanoparticles, and allowing prolonged and controlled delivery [127,129]. The most commonly used include lipid nanoparticles, such as nanoemulsions, solid lipid nanoparticles (SLN), lipid nanostructured carriers (NLC), liposomes and niosomes, polymer nanoparticles, metal nanoparticles, nanocrystals, and nanospheres [130]. The selection of an appropriate nanocarrier for topical molecules delivery is highly dependent on the purpose of the study, e.g., enhancing permeation (transdermal delivery) or targeted delivery to skin organelles, and the nature of the active compounds that may be encapsulated. The compatibility of the nature of the nanocarriers with the structure of the skin should also be assessed, which will help in more precise penetration of drugs through the skin (Figure 5) [126,127,131].

Carbone et al. noted that the combined use of EOs (*Rosmarinus officinalis*, *Lavandula* × intermedia “Sumian”, *Origanum vulgare* subsp. hirtum) and clotrimazole delivery via lipid nanoparticles showed potential synergistic effects against skin infections caused by *Candida albicans*, *Candida krusei*, and *Candida parapsilosis.* The increase in antifungal activity of clotrimazole-loaded nanoparticles prepared from *Lavandula* or *Rosmarinus* confirmed that NLCs containing EOs are a promising strategy for improving the efficacy of drugs against topical candidiasis [132].

Another study examined the use of NLCs used to deliver essential oils of *Rosmarinus officinalis*, *Lavandula* × intermedia ‘Sumian’, *Origanum vulgare* subsp. Hirtum, and *Thymus capitatus*. The oils showed antioxidant activity in the DPPH test and anti-inflammatory activity in RAW 264 cells after treatment with lipopolysaccharide (LPS) to induce nitric oxide (NO) production. The *Lavandula* and *Rosmarinus*-NLCs proved to be the most biocompatible formulations up to a concentration of 0.1% (*v*/*v*), and were able to induce dose-dependent anti-inflammatory activity in the order *Lavandula* > *Rosmarinus* ≥ *Origanum* [133].

In turn, Vanti et al. found the most active EOs loaded onto propylene glycol nanoparticles to be *Origanum onites*, comprising carvacrol (66.0%), p-cymene (7.9%), ϒ-terpinene (4.9%), and borneol (2.8%), and *Satureja thymbra*, comprising carvacrol (46.0%), ϒ-terpinene (19.7%), p-cymene (7.6%), β-caryophyllene (7.0%), and α-terpinene (5.1%); these were all effective against *Staphylococcus aureus* and *Pseudomonas aeruginosa*, and the fungal strains *Candida albicans* and *Candida krusei* [134]. Other examples of studies are presented in Table 4.

## 10. Patented Compositions of Essential Oils and Their Role in Skin Lesions

It is worth noting that EOs derived from various species of the Lamiaceae family can be applied topically to the skin, offering numerous benefits. A distinction can be made between natural products or ingredients directly isolated from natural products, and semi-synthetic ones created through structural modification of their natural compounds [150,151]. Their use in terms of various applications can be recorded as a patent [152,153,154]. Table 5 below shows patent applications for products related to skin applications containing essential oils isolated from various species belonging to the Lamiaceae family.

## 11. Conclusions

The review compiles a range of studies assessing the activities of various Lamiaceae EOs beneficial to skin. These oils demonstrate wound-healing, antiaging, anti-melanogenic, and anti-cancer effects, which can be attributed to their antioxidant and anti-inflammatory activities. More precisely, they appear to inhibit relevant ROS and proinflammatory cytokine production and activity, enhance tissue remodeling and re-epithelialization, and inhibit collagenase, elastase, and tyrosinase. They are also toxic to skin cancer cells. Furthermore, recent years have witnessed the development of more effective topical formulations, including nanoparticles. Some formulations based on Lamiaceae EOs have been patented. This review highlights the great potential of Lamiaceae EOs as dermatological agents.

## Figures and Tables

**Table 1:** The anti-inflammatory effects of the Lamiaceae essential oils in vitro.

Tested Plant	Plant Part	Number of Identified Compounds (the Main Constituents > 5%)	Cell Line	Tested Essential Oil Concentrations	Effects	Ref.
*Glechoma hederacea* L.	aerial parts	29 compounds (trans-3-pinanone, β-caryophyllene, 4,5,6,7-tetrahydro-5-isopropenyl-3,6-betadimethyl-6-alpha-vinylbenzofuran)	RAW 264.7 macrophages stimulated with LPS	5–20 μg/mL	suppress NO production, regulate expression of iNOS, COX-2, and HO-1, and TNF-α	[55]
*Lavandula angustifolia* L.	-	(linalyl acetate, linalool, β-caryophyllen, trans-βocimene, lavandulyl acetate)	Murine brain endothelial bEnd.3 cells stimulated with TNF-α	0.01%	inhibition of TNF-α-induced NF-κB activation	[56]
*Lavandula angustifolia* L.	whole plant	71 compounds (linalool, terpinene-4-ol, α-terpineol, linalyl acetate)	THP-1 human monocyte/macrophage stimulated with LPS	100 μL of DMSO was added to 900 μL of essential oil in a final volume of 1 mL. The emulsions were diluted with phosphate-buffered saline 500-fold	decreased IL-6, IL-1β, and IL-8 expression	[57]
*Monarda* *Didyma* L.	flowering aerial parts	20 compounds (1-octen-3-ol, p-cymene, γ-terpinene, thymol methyl ether, carvacrol methyl ether, thymol, carvacrol)	U937 cells stimulated with LPS	0.5 μL/mL	decreased expression of IL-6	[58]
*Ocimum basilicum* L.	whole plant	25 compounds (the distillate fraction contained estragole, methyl eugenol, α-bergamotene, carotol, α-cadinol)	RAW 264.7 macrophages stimulated with LPS	20 µg/mL	the distillate fraction suppressed the production of NO and iNOS, and expression of TNF-α, IL-1β, and IL-6	[59]
*Ocimum sanctum* L.	leaves	-	Lymphocytes stimulated with LPS	250 μg/mL	downregulation of MMP-9 expression	[60]
*Origanum vulgare* L.	-	32 constituents (carvacrol, thymol, p-cymene)	human keratinocytes NCTC 2544 treated with interferon-gamma (IFN-γ) and histamine (H)	25 μg/mL	reduction of ROS, ICAM-1, iNOS, and COX-2	[61]
*Pogostemon plectranthoides* Desf.	leaf	37 compounds (cyclosativene, caryophyllene oxide, 1-epi-cubenol, eudesma-4(15), 7-dien-1-β-ol, mustakone)	human red blood cell	62.5–1000 μg/mL	cell membrane stabilization activity	[62]
*Rosmarinus officinalis* L.	whole plant	-	THP-1 human monocyte/macrophage stimulated with LPS	5 μg/mL	increased level of IL-10 expression	[63]
*Salvia officinalis* L.	aerial parts	25 compounds, (1,8-cineole, camphor, β-pinene, α-terpineol, α-pinene)	RAW 264.7 macrophages stimulated with LPS	0.16–1.25 µL/mL	inhibited NO production	[64]
*Salvia officinalis* L.	leaves	24 compounds (camphene, 1,8-cineole, α-thujone, camphor, bornyl acetate)	RAW 264.7 macrophages stimulated with LPS	50–500 μg/mL	reduced NO and NF-κB production	[65]
*Thymus albicans* L.	flowering parts	35 compounds (1,8-cineole, linalool, borneol)	RAW 264.7 macrophages stimulated with LPS	0.32–0.64 μL/mL	reduced the production of nitrites, an NO-derived sub-product, and iNOS protein levels	[66]
*Thymus camphoratus* L.	flowering aerial parts	60 compounds (α-pinene, camphene, 1,8-cineole, linalool, borneol)	RAW 264.7 macrophages stimulated with LPS	0.16–0.32 μL/mL	inhibitory effects towards NO production, inhibiting the expression of iNOS and COX-2	[67]
*Thymus zygis* L.	aerial parts	41 compounds (p-cymene, thymol, carvacrol, γ-terpinene, linalool)	RAW 264.7 macrophages stimulated with LPS	0.08–0.64 µL/mL	inhibition of NO production	[68]

**Table 2:** The anti-inflammatory effects of the *Lamiaceae* essential oils in vivo.

Tested Plant	Plant Part	Number of Identified Compounds (the Main Constituents > 5%)	Animals	Tested Essential Oil Concentrations/Types of Administration (Oral/Topical)	Effects	Ref.
*Agastache rugosa* Gronov.	leaves	37 compounds (p-allylguaiacol/eugenol, patchouli alcohol, pogostone)	Rats with adjuvant arthritis	100 mg/kg (oral administration)	inhibition of the expression of IL-1, IL-6, TNF-α, and COX-2	[69]
*Hyptis spicigera* Lam.	aerial parts	14 compounds (α-pinene, β-pinene, 1,8-cineole, β-caryophyllene)	Swiss mice	1000 mg/kg (oral administration)	temperature of the hind paw was reduced; edema was diminished	[70]
*Lavandula* *Augustifolia* L.	-	28 compounds (D-limonene, linalyl acetate, linalool)	Swiss mice	50 μL/ear (topical treatment)/ 0.6 g/kg (oral treatment)	inhibition of paw edema induced by carrageenan and by croton oil	[71]
*Lavandula angustifolia* L.	leaves and stem	27 compounds (1,8-cineole, borneol, camphor, limonene, camphene)	Swiss mice	0.25, 0.5, and 1 mg/ear (topical administration) 75, 100, and 250 mg/kg (oral administration)	topical treatment reduced edema formation, MPO activity, and NO production in croton-oil-induced ear edema model or carrageenan-induced paw edema model/oral treatment reduced edema formation, MPO activity, and NO production	[72]
*Lavandula angustifolia* L.		24 compounds (D-limonene, α-pinene, linalool, linalyl acetate, isobornyl acetate, benzylacetone)	BALB/c mice 12-O-tetradecanoyl phorbol-13-acetate (TPA)-induced mice models	100 µg/mL (topical administration)	decreased the production of TNF-α, NF-κB, and IL-6	[73]
*Lavandula angustifolia* L.	aerial parts	54 compounds (γ-terpineol, lavandulyl propionate)	Rats with adjuvant arthritis	100 mg/kg (oral administration)	inhibition of the expression of IL-1, IL-6, TNF-α, and COX-2	[69]
*Lavandula stoechas* L.	aerial parts	21 compounds (β-pinene, 1,8-cineole)	Swiss albino mice	200 and 20 mg/kg (oral administration) 82 and 410 mg/kg (topical administration)	reduced carrageenan-induced paw edema/ reduced acute ear edema	[74]
*Lavandula stoechas* L.	aerial parts	28 compounds (1,8-cineole, trans-α-necrodyl acetate, E-caryophyllene, trans-α-necrodol, lavandulol)	Swiss albino mice	200 mg/kg (oral administration)	inhibition of carrageenan-induced rat paw oedema	[75]
*Melissa officinalis* L.	leaves	(nerol, citral, isopulegol)	Wistar rats	200, 400 mg/kg (oral administration)	reduction in edema induced by carrageenan	[76]
*Mentha haplocalyx* L.		32 compounds (p-cymene, D-limonene, γ-terpinene, α-isomenthone, L-menthone, DL-menthol)	BALB/c mice 12-O-tetradecanoyl phorbol-13-acetate (TPA)-induced mice models	100 µg/mL (topical administration)	decreased the production of TNF-α, NF-κB, IL-6, and COX-2	[73]
*Mentha piperita* L.		28 compounds (menthone, isomenthone, menthol, trans-anethole)	Charles River Wistar rats	125–500 mg/kg (oral administration)	inhibited paw edema induced by carrageenan	[77]
*Mentha piperita* L.	leaves	51 compounds (neomenthol, menthol, menthy acetate)	ICR mice	200, 400 and 800 mg/ear (topically treatment)	inhibition of paw edema induced by croton oil	[78]
*Mentha spicata* L. subsp. crispata		28 compounds (menthone, menthol, carvone)	Charles River Wistar rats	125–500 mg/kg (oral administration)	inhibited paw edema induced by carrageenan	[77]
*Mentha suaveolens* L.		20 compounds (piperitenone oxide)	Charles River Wistar rats	1125–500 mg/kg (oral administration)	inhibited paw edema induced by carrageenan	[77]
*Ocimum* *basilicum* L.	leaves	14 compounds (linalool, estragole)	Swiss albino mice	100 µg/mL (topical administration)	reduced paw edema induced by carrageenan and dextran	[79]
*Ocimum kilimandscharicum* L.	leaves	45 compounds (limonene, 1,8 cineole, camphor)	Swiss mice	30 and 100 mg/kg (oral administration)	inhibited carrageenan-induced pleurisy	[80]
*Ocimum selloi* L.	leaves	9 compounds (methyl chavicol, E-anethole)	Swiss mice	30–300 mg/kg (oral administration)	significantly prevented paw edema, mechanical hyperalgesia, and cold hyperalgesia after carrageenan model	[81]
*Origanum compactum* L.	aerial parts	11 compounds (p-cymene, β-pinene, carvacrol, thymol)	Wistar rats	100 mg/kg (oral administration)	inhibition of paw edema induced by carrageenan	[82]
*Perilla frutescens* (L.) Britton		20 compounds (linalool, 2-pyrimidinamine, 2-hexanoylfuran, β-caryophyllene)	BALB/c mice 12-O-tetradecanoyl phorbol-13-acetate (TPA)-induced mice models	100 µg/mL (topical administration)	decreased the production of TNF-α, NF-κB, IL-6, and COX-2	[73]
*Perilla frutescens* (L.) Britton	leaves	24 compounds (β-caryophyllene, linalool)	Rats with adjuvant arthritis	100 mg/kg (oral administration)	inhibited the expression of IL-1, IL-6, TNF-α, and COX-2	[69]
*Pogostemon* *cablin* (Blanco) Benth.		14 compounds (α-guaiene, α-bulnesene, seychellene, patchouli alcohol)	BALB/c mice 12-O-tetradecanoyl phorbol-13-acetate (TPA)-induced mice models	100 µg/mL (topical administration)	decreased the production of TNF-α, NF-κB and COX-2	[73]
*Pogostemon cablin* (Blanco) Benth.	leaves	35 compounds (p-allylguaiacol/eugenol, patchouli alcohol, pogostone)	Rats with adjuvant arthritis	100 mg/kg (oral administration)	Inhibited the expression of IL-1, IL-6, TNF-α, and COX-2	[69]
*Rosmarinus offcinalis* L.	leaves	46 compounds (levo verbenone, chavibetol, borneol, (+)-2-bornanone, eucalyptol)	Rats with adjuvant arthritis	100 mg/kg (oral administration)	Inhibited the expression of IL-1, IL-6, TNF-α, and COX-2	[69]
*Rosmarinus officinalis* L.		23 compounds (D-limonene, α-pinene, linalool)	BALB/c mice 12-O-tetradecanoyl phorbol-13-acetate (TPA)-induced mice models	100 µg/mL (topical administration)	decreased the production of TNF-α, NF-κB, and IL-6	[73]
*Salvia japonica* L.	aerial parts	47 compounds (L-α-pinene, linalool, (+)-2-bornanone, benzyl acetate, triacetin, terpenyl acetate)	Rats with adjuvant arthritis	100 mg/kg (oral administration)	inhibited the expression of IL-1, IL-6, TNF-α, and COX-2	[69]
*Scutellaria baicalensis* Georgi.		44 compounds (o-cymene, curcumene, (Z,E)-α-farnesene, γ-muurolene)	BALB/c mice 12-O-tetradecanoyl phorbol-13-acetate (TPA)-induced mice models	100 µg/mL (topical administration)	decreased the production of TNF-α, NF-κB, IL-6, and COX-2	[73]
*Stachys lavandulifolia* Vahl.	aerial parts	(–)-α-bisabolol, bicyclogermacrene	Swiss mice	25 or 50 mg/kg (oral administration)	reduced pro-inflammatory cytokine IL-1β	[83]
*Thymus* *algeriensis* Boiss. & Reut.	aerial parts	9 compounds (borneol, thymol, carvacrol)	rats	150 mg/kg (oral administration)	inhibited paw edema induced by carrageenan	[84]
*Thymus fontanesii* Boiss. & Reut.	aerial parts	24 compounds (p-cymene, γ-terpinene, carvacrol)	mice	50 mg/kg and 100 mg/kg (oral administration)	inhibited paw edema induced by carrageenan	[85]
*Thymus vulgaris* L.	aerial parts	66 compounds (p-cymene, γ-terpinene, thymol)	Swiss albino mice	400 mg/kg (oral administration)	reduction in edema induced by carrageenan	[86]
*Thymus vulgaris* L.	-	25 compounds (p-cymene, γ-terpinene, carvacrol)	Swiss mice	100, 10 and 2 mg/kg (topical administration)	inhibited paw edema induced by croton oil	[87]
*Zataria multiflora* Boiss.	-	29 compounds (p-cymene, γ-terpinene, thymol, carvacrol)	BALB/c mice	1–2% (topical administration)	decreased the expression of IL-1β and TNF-α	[88]

**Table 3:** The cytotoxic effects of essential oils derived from the Lamiaceae family on melanoma cells.

Tested Plant	Plant Part	Number of Identified Compounds (the Main Constituents)	Cell Line	Essential Oil Concentrations	Ref.
*Cantinoa stricta* (Benth.) Harley & J. F. B. Pastore	flowers	46 compounds (α-pinene, β-pinene, limonene + β-phellandrene, spathulenol, caryophyllene oxide)	UACC-62	TGI = 25.19 µg/mL	[111]
*Cedronella canariensis* (L.) Webb & Berthel	aerial parts	61 compounds (β-pinene, pinocarvone)	A375	IC_50_ = 4.3 µg/mL	[112]
*Cunila angustifolia* Benth.	leaves	17 compounds (menthone, isomenthol, pulegone)	SK-Mel-28	IC_50_ = 279.9 µg/mL	[113]
*Lavandula stoechas* L.	aerial parts	21 compounds (β-pinene, 1,8-cineole)	MV3	IC_50_ = 0.06 µL/mL	[74]
*Mentha piperita* L.	aerial parts	-	A-375	IC_50_ = 0.4 µL/mL	[114]
*Ocimum* *basilicum* L.	leaves	linalool and isoeugenol	FemX	IC_50_ = 96.72 µg/mL	[115]
*Ocimum basilicum* L.	aerial parts	-	A-375	IC_50_ = 0.36 µL/mL	[114]
*Origanum vulgare* L.	aerial parts	-	A-375	IC_50_ = 0.09 µL/mL	[114]
*Pogostemon deccanensis* Desf.	aerial parts	47 compounds (ethanone, 1-(2,4,6-trihydroxyphenyl)-, epi-cadinol, benzofuran, 6-ethenyl-4,5,6,7-tetrahydro-3,6-dimethyl-5-isopropenyl-, trans-)	B16F1	2 µg/mL—2.1% survival ratio	[116]
*Rosmarinus officinalis* L.	leaves	-	A-375	IC_50_ = 0.24 µL/mL	[114]
*Salvia aurea* L.	aerial parts	35 compounds (aromadendrene, α-amorphene, caryophyllene oxide, elemenone, aristolone)	M14	IC_50_ = 12.5 µg/mL	[117]
*Salvia aurea* L.	aerial parts	35 compounds (aromadendrene, α-amorphene, caryophyllene oxide, elemenone, aristolone)	A2058	IC_50_ = 21.2 µg/mL	[117]
*Salvia aurea* L.	aerial parts	35 compounds (aromadendrene, α-amorphene, caryophyllene oxide, elemenone, aristolone)	A375	IC_50_ = 15.9 µg/mL	[117]
*Salvia judaica* Boiss	aerial parts	45 compounds (tetradecanoic acid, caryophyllene oxide, α-copaene)	M14	IC_50_ = 11.6 µg/mL	[117]
*Salvia Judaica* Boiss	aerial parts	45 compounds (tetradecanoic acid, caryophyllene oxide, α-copaene)	A2058	IC_50_ = 19.4 µg/mL	[117]
*Salvia Judaica* Boiss	aerial parts	45 compounds (tetradecanoic acid, caryophyllene oxide, α-copaene)	A375	IC_50_ = 14.4 µg/mL	[117]
*Salvia officinalis* L.	whole plant	14 compounds (1,8-cineole, α-thujone, β-thujone, camphor, γ-muurolene)	A375	IC_50_ = 10.7 µg/mL	[118]
*Salvia officinalis* L.	whole plant	10 compounds (α-thujone, β-thujone, γ-elemene, γ-muurolene, sclareol)	M14	IC_50_ = 8.2 µg/mL	[118]
*Salvia officinalis* L.	whole plant	10 compounds (α-thujone, β-thujone, γ-elemene, γ-muurolene, sclareol)	A2058	IC_50_ = 11.7 µg/mL	[118]
*Salvia officinalis* L.	aerial parts	14 compounds (β-pinene, eucalyptol, α-thujone, camphene, p-thymol, caryophyllene)	A375	50 µg/mL—39% inhibition ratio	[119]
*Salvia verbenaca* L.	aerial parts	76 constituents (hexahydrofarnesyl acetone, hexadecanoic acid)	M14	IC_50_ = 8.1 µg/mL	[120]
*Salvia viscosa* Jacq.	aerial parts	31 compounds (β-copaen-4-α-ol, caryophyllene oxide, α-cubebene, carvacrol)	M14	IC_50_ = 13.3 µg/mL	[117]
*Salvia viscosa* Jacq.	aerial parts	31 compounds (β-copaen-4-α-ol, caryophyllene oxide, α-cubebene, carvacrol)	A2058	IC_50_ = 23.6 µg/mL	[117]
*Salvia viscosa* Jacq.	aerial parts	31 compounds (β-copaen-4-α-ol, caryophyllene oxide, α-cubebene, carvacrol)	A375	IC_50_ = 16.2 µg/mL	[117]
*Satureja hortensis* L.	aerial parts	18 compounds ((+)-4-carene, γ-terpinene, o-cymene, thymol, carvacrol)	A375	IC_50_ = 22.27 µg/mL	[121]
*Stachys annua* L.	aerial parts	53 compounds (phytol, germacrene D, spathulenol, bicyclogermacrene)	A375	IC_50_ = 37.2 µg/mL	[122]
*Thymus munbyanus* Boiss & Reuth	flowers	103 compounds (1,8-cineole, camphor, borneol)	A375	IC_50_ = 46.95 µg/mL	[123]
*Thymus vulgaris* L.	aerial parts	8 compounds (γ-terpinene, p-thymol, caryophyllene)	A375	50 µg/mL—17.5% inhibition ratio	[119]

IC_50_—half-maximal inhibitory concentration—concentration that inhibited cell growth by 50%. TGI—total growth inhibition—concentration that inhibited cell growth by 100%.

**Table 4:** Nanoparticles with essential oils from the Lamiaceae family.

Tested Plant	Chemical Components of Essential Oils	Type of Nanoparticles	Activities	Effect	References
*Ocimum basilicum*	eugenol and caryophyllene	chitosan nanoparticles	antibacterial and antibiofilm activity	*Staphylococcus aureus*	[135]
*Thymus* sp.	Thymol and carvacrol	chitosan nanoparticles	antimicrobial activity	*Staphylococcus aureus*	[136]
*Mentha* sp.	Menthol, menthone, menthyl acetate, piperitone, limonene, and 1,8-cineole	hydroxyapatite nanoparticles	antimicrobial activity	*Staphylococcus aureus*, *Pseudomonas aeruginosa,* or the fungal strain *Candida parapsilosis*	[137]
*Lavendula* sp.	-	nanostructured lipid carriers (NLCs)	wound-healing activities		[138]
*Rosmarinus officinalis* L.	-	silver nanoparticles	antimicrobial and wound-healing activity	*Staphylococcus aureus*	[139]
*Zataria multiflora* Boiss.	-	solid lipid nanoparticles	anti-cancer	anticancer efficacy of the essential oil against melanoma cancer (A-375) cells with 75 μg/mL	[140]
*Mentha piperita* L.	-	chitosan nanoparticles	antioxidant and antimicrobial activities	enhanced antibacterial activity with MBC values of 0.57 and mg·mL^−1^ against *S. aureus*; antioxidant activities were improved by about 2.4-fold in DPPPH test	[141]
*Satureja khuzistanica* Jamzad	carvacrol	chitosan nanoparticles	antibacterial activities	activities on *Pseudomonas aeruginosa*, *Staphylococcus aureus*, and *Staphylococcus epidermidis* strains	[142]
*Origanum vulgare* L.	-	gold nanoparticles	antioxidant, antimicrobial properties	significant bactericidal and antioxidant activities, the most sensitive microorganisms being *S. aureus* and *C. albicans*, better tolerated by normal human dermal fibroblast cells, while the melanoma cancer cells are more sensitive	[143]
*Origanum vulgare* L.	-	ZnO nanoparticles	antioxidant activity	excellent antioxidative properties in DPPH test	[144]
*Satureja hortensis* L.	-	iron nanoparticles (FeNPs)	antimicrobial activity	possessed higher antimicrobial properties against selected pathogenic microorganisms, *S. aureus, P. aeruginosa*, and *C. albicans*	[145]
*Origanum vulgare* L.	o-cymene/m-cymene, terpinolene, carvacrol, -terpinene	chitosan—alginate nanoparticles	antimicrobial activity	possessed strong antimicrobial activity against *S. aureus*, *P. aeruginosa*, and *C. albicans*	[146]
*Thymus capitatus* L. and *Origanum vulgare* L.	carvacrol, thymol	chitosan nanoparticles	antimicrobial activity	exhibited enhanced bactericidal activity against *S. aureus*	[147]
*Thymus vulgaris* L.	*p*-cymene, thymol, α-terpineol and linalool	archaeolipids carriers (NAC)	antioxidant, anti-inflammatory, and antibiofilm activity	exhibited enhanced activity against *P. aeruginosa*	[148]
*Lavandula angustifolia* L.	-	silver nanoparticles	antimicrobial and wound-healing activity	excellent bactericidal properties against *S. aureus*	[149]

**Table 5:** Patent information on products based on essential oils from the Lamiaceae family applied to skin lesions.

The Active Ingredient from the Lamiaceae Family	Application	Patent Number	Year
Mentha camphor oil, *Lavandula angustifolia* oil	Skin pruritus, allergic dermatitis, eczema	CN106420937A	2017
*Melissa* oil	An organic skin moisturizer	US 8,986,752 B1	2015
*Origanum compactum* oil	Treatment of keratoses	US 9,040,103 B2	2015
Peppermint oil	Inflammation of skin	US 9,180,146 B2	2015
*Monarda fistulosa* and/or *Monarda didyma* oil	Inflammation of skin	US 2016/0213727 A1	2016
*Ocimum americanum* oil, *Mentha pulegium* oil	Cosmetic application	WO2017112998A1	2017
Oregano oil, Thyme oil	Bacterial and fungal infections, and oxidative stress	WO2016187422A1	2016
*Origanum compactum oil*	Therapeutic treatment of actinic keratoses	EP2538933B1	2016
*Origanum compactum* oil	Treatment of malign keratosis	EP2538933A2	2016
Rosemary oil, peppermint oil	A hand and body skincare cream	US7887853B1	2011

## Data Availability

Not applicable.
